# Extraction of Carotenoids and Fat-Soluble Vitamins from *Tetradesmus Obliquus* Microalgae: An Optimized Approach by Using Supercritical CO_2_

**DOI:** 10.3390/molecules24142581

**Published:** 2019-07-16

**Authors:** Laura Chronopoulou, Chiara Dal Bosco, Fabrizio Di Caprio, Letizia Prosini, Alessandra Gentili, Francesca Pagnanelli, Cleofe Palocci

**Affiliations:** 1 Chemistry Department, University of Rome La Sapienza, 00185 Rome, Italy; 2 CIABC, University of Rome La Sapienza, 00185 Rome, Italy

**Keywords:** microalgae, *Scenedesmus*, supercritical fluid extraction, carotenoids, fat-soluble vitamins, antioxidants

## Abstract

In recent years, great attention has been focused on rapid, selective, and environmentally friendly extraction methods to recover pigments and antioxidants from microalgae. Among these, supercritical fluid extraction (SFE) represents one of the most important alternatives to traditional extraction methods carried out with the use of organic solvents. In this study, the influence of parameters such as pressure, temperature, and the addition of a polar co-solvent in the SFE yields of carotenoids and fat-soluble vitamins from *T. obliquus* biomass were evaluated. The highest extraction of alpha-tocopherol, gamma-tocopherol, and retinol was achieved at a pressure of 30 MPa and a temperature of 40 °C. It was observed that overall, the extraction yield increased considerably when a preliminary step of sample pre-treatment, based on a matrix solid phase dispersion, was applied using diatomaceous earth as a dispersing agent. The use of ethanol as a co-solvent, under certain conditions of pressure and temperature, resulted in selectively increasing the yields of only some compounds. In particular, a remarkable selectivity was observed if the extraction was carried out in the presence of ethanol at 10 MPa and 40 °C: under these conditions, it was possible to isolate menaquinone-7, a homologous of vitamin K2, which, otherwise, cannot not recovered by using traditional extraction procedures.

## 1. Introduction

Aquatic species are promising sources of products for the fine chemicals industry, and this has aroused a growing interest toward such organisms for several applications such as the production of biofuels, the extraction of food additives or active ingredients for cosmetic formulations [1,2,3]. In particular, algae represent an attractive source for the extraction of vitamin K, carotenoids, and other fat-soluble vitamins.

Vitamin K is a family of structurally similar chemical compounds including phylloquinone (vitamin K1), which occurs in green plants, and menaquinones (vitamin K2 vitamers), which are predominantly of microbial origin [4,5]. Besides acting as a cofactor for the enzyme γ-glutamylcarboxylase, recent research has shown that vitamin K can protect against intracellular oxidative stress and cognitive decline [6,7,8,9]. Regarding the vitamin K content in common macroalgae, extremely variable concentrations of phylloquinone have been observed [10,11], however, it was not detected in *P. tricornutum* [11], while its concentration reached 750 µg/100 g in *Sargassum muticum* (commonly known as Japanese wireweed), which is a significantly higher value than that observed in terrestrial plants [10]. To the best of our knowledge, no information on the distribution of menaquinones has so far been reported. 

Carotenoids are tetraterpenoids with numerous biological functions synthesized by plants, algae, fungi, and bacteria. They are essential for photosynthesis and, in general, for life in the presence of oxygen. Due to their chemical structure, based on a long chain of conjugated double bonds, these micronutrients are highly lipophilic, variously colored, and exhibit antioxidant properties [12]. Due to changes in lifestyle and the rising health consciousness of the average population, the demand for nutrient-rich supplements with health benefits has risen substantially. Carotenoids also have various medicinal properties and are widely used as preventatives against diseases such as cancer, diabetes, and cataracts [13]. Carotenoids are also currently used in food supplements, cosmetics, and pharmaceuticals [14]. 

Hydrocarbon carotenoids are referred to as carotenes, while their oxygenated derivatives are known as xanthophylls. Within the latter group, the oxygen atom may be present in the form of hydroxyl groups (as in the case of lutein) or as keto groups (as in the case of canthaxanthin) or as a combination of both (as in astaxanthin) [15]; other oxygenated functional groups occurring in xanthophylls are the epoxy group and carboxylic group. Moreover, all carotenoids having a polyene chain with 11 carbon atoms, and at least one unsubstituted β-ionone ring contributes to the synthesis of vitamin A by means of their conversion into retinol. Increasingly restrictive legislation regarding the origin of food preservatives (e.g., antioxidants and antimicrobials), coupled with the growing demand for natural compounds, have renewed the interest in biomass as a potential source for such compounds rather than resorting to chemical synthesis [16,17]. The best candidates for carotenoid biosynthesis are microalgae because they have many useful features from an industrial point of view: a high surface-to-volume ratio; high growth rates; different metabolic pathways; environmental adaptability; and the simplicity of screening and genetic manipulation [10]. Typical maximum growth rates of microalgae are between 0.5 and 2 d^−1^ that correspond to the duplication times of a few hours, which is much higher than the growth rates of terrestrial plants. Although microalgae are photosynthetic microorganisms, they can also grow in mixotrophic and heterotrophic conditions [18]. Microalgae generally have an optimal pH between 7–9 and an optimal temperature between 25–30 °C. Some carotenoids are synthesized by microalgae as primary metabolites to protect the photosystems from photodamage caused by excessive light exposition and to enlarge the harvesting spectrum of light (e.g., lutein and fucoxanthin). Others carotenoids such as astaxanthin and β-carotene are accumulated as secondary metabolites under stress conditions (e.g., nutrients starvation, osmotic shock, high temperature) to protect the cells from oxidative stresses [14,19]. Among the various species of microalgae, *Tetradesmus obliquus* (generally known as *Scenedesmus obliquus*), an ubiquitous microorganism in lakes and freshwater rivers, is one of the most studied strains for large scale industrial applications, thanks to its ability to grow well in the non-optimal conditions typical of industrial outdoor plants [20]. *T obliquus* can be cultivated with biomass productivities until 2 g/L d in photoautotrophic conditions [21] and until 6 g/L d in heterotrophic conditions [18]. This microalga appears to be a promising source of carotenoid-rich extracts [22]. However, to date, *T. obliquus* is still not exploited for the industrial production of carotenoids or vitamins [19]. One relevant limitation comes from the absence of extraction processes that are sufficiently selective and efficient [23]. Among the most widely used organic solvents for carotenoid extraction, there is hexane (particularly effective for carotenes), ethanol (advantageous for xanthophylls), and acetone. However, the use of organic solvents for extraction is not the preferred way to produce healthy foods due to the toxicity of solvent residues in food as well as the issues of environmental pollution. The development of an efficient extraction technique for the isolation of pharmaceutical compounds from natural resources is necessary. 

Extraction using supercritical fluids (SFE) is currently considered an important green alternative to traditional methods. The properties of supercritical fluids (SFs) can be considered as intermediate between the ones of liquids and gases. Similarly to gases, SFs are highly compressible, but have high densities comparable to those of liquids. The combination of some of the properties of liquids with those of gases provides supercritical fluids with some very interesting features. For example, supercritical fluids can effuse through solid materials like a gas, but can also act like a liquid and dissolve substances. In SFE, the organic phase used in typical solid–liquid extractions (SLE) is substituted by a supercritical fluid. The manipulation of both the temperature and pressure of the fluid can solubilize the substance of interest in a complex matrix and selectively extract it. Compared to SLE, SFE is indeed simpler, faster, and more efficient but without consuming large quantities of organic solvents, which are both expensive and potentially harmful. Other immediate advantages of SFE compared to traditional extraction techniques are process flexibility due to the continuous modulation of the solvent power/selectivity of the supercritical fluid and the elimination of polluting organic solvents, which also prevents expensive post-processing of the extracts for solvent removal. CO_2_ is the most commonly used supercritical fluid thanks to its non-toxicity, chemical inertia, low cost, and most importantly, low critical values. Its low critical temperature (below 32 °C) makes CO_2_ ideal for the extraction of thermolabile compounds. For these reasons, the use of CO_2_ as an extraction solvent has been successfully reported in the literature for the isolation of many compounds from various sources [24,25,26,27,28]. For example, supercritical CO_2_ has been tested for the extraction of carotenoids and triglycerides from microalgae such as *Hematococcus pluvialis*, *Scenedesmus* sp., and *Chlorella* sp. in different previous works, by mainly using ethanol as the co-solvent, as reported in a recent review [29]. However, there is scarce information about the co-extraction of vitamins and carotenoids from microalgae. In this study, the possibility of extracting carotenoids and fat-soluble vitamins from *T. obliquus* by means of CO_2_ in the supercritical phase was evaluated. The effect of several parameters such as the CO_2_ physical variables, the addition of co-solvents (methanol and limonene), and an inert dispersing phase on the recovery of different carotenoids and fat-soluble vitamins was investigated. The supercritical fluid extraction was also compared in terms of yield and selectivity with conventional extraction methods. 

## 2. Results and Discussion

### 2.1. Extraction via Matrix Solid-Phase Dispersion

According to the methodology reported in the literature [30,31], the HPLC-MS analysis of the extracts obtained by the matrix solid-phase dispersion (MSPD) showed that the most abundant carotenoids and fat-soluble vitamins in the algal biomass were lutein and α-tocopherol, respectively. The MSPD extraction, applied individually in accordance with that described in Section 3.4, allowed 10 different compounds to be isolated and identified via HPLC-MS. 

### 2.2. Supercritical CO_2_ (SCCO_2_) Extraction 

#### 2.2.1. Evaluation of the Optimal Extraction Time

A series of preliminary SFE was performed on samples of *T. obliquus* at constant pressure (P) and temperature (T) values and by varying the extraction time between 1 and 4 h in order to determine its optimal value. In particular, by using a P_CO2_ of 30 MPa and a T_CO2_ of 50 °C, the percentage of extracted carotenoids was evaluated as a function of the incubation time. The 90-min extraction time was selected as the most appropriate one since, at higher incubation times, no increase in the amount of extracted material was recorded. Therefore, all subsequent extractions in the static approach were carried out for 90 min. For operations in the dynamic mode, an initial static extraction time of 90 min was used, followed by a dynamic extraction step for 10 min. In both cases, at the end of the process, CO_2_ was withdrawn from the cell and the extract was recovered in 2 mL of ethanol.

#### 2.2.2. Addition of Diatomaceous Earth as a Dispersing Phase

Before extraction, the algal biomass was mixed with diatomaceous earth in a 1:10 *w*/*w* proportion, as reported in the experimental section. This sample preparation was selected with the aim of obtaining a better yield and extraction reproducibility (data not shown). Indeed, mixing with diatomaceous earth can help to break the cell walls and membranes of the microalgae, exposing the cellular content to the action of the extracting fluid as well as increasing the contact area between the sample and the solvent.

### 2.3. Evaluation of the Influence of Pressure and Temperature on the Recovery of Carotenoids from T. Obliquus

#### 2.3.1. Extraction Conditions and Extraction Variables

For all of the SFE and MSPD extraction procedures, a fixed quantity of diatomaceous earth/microalga mixture (equal to 0.200 g of sample, of which 0.01818 g was *T. obliquus* biomass) was used. Each experiment was repeated at least twice. Three different CO_2_ pressure values were selected during the extractions (25, 30, and 35 MPa, respectively) and for each pressure, the extractions were performed at three different temperatures (40, 50, and 60 °C). The pressure and temperature values were selected on the basis of the literature data and taking into account the thermolability of the carotenoids. In general, as expected, it was observed that as the pressure of the supercritical CO_2_ increases at constant temperature, the extraction yields increase as a consequence of an increase in the solvent power. In contrast, as the temperature increases at constant pressure, the solvent power of CO_2_ decreases, and therefore carotenoid extraction yields are reduced.

**Figure 1:**
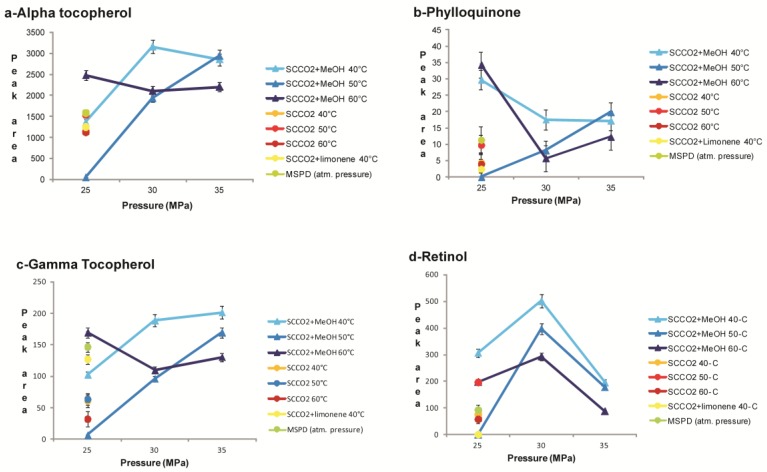
Extraction yields (average peak area ± standard error) of (**a**) alpha tocopherol, (**b**) phylloquinone, (**c**) gamma tocopherol, and (**d**) retinol obtained with SFE and MSPD.

**Figure 2:**
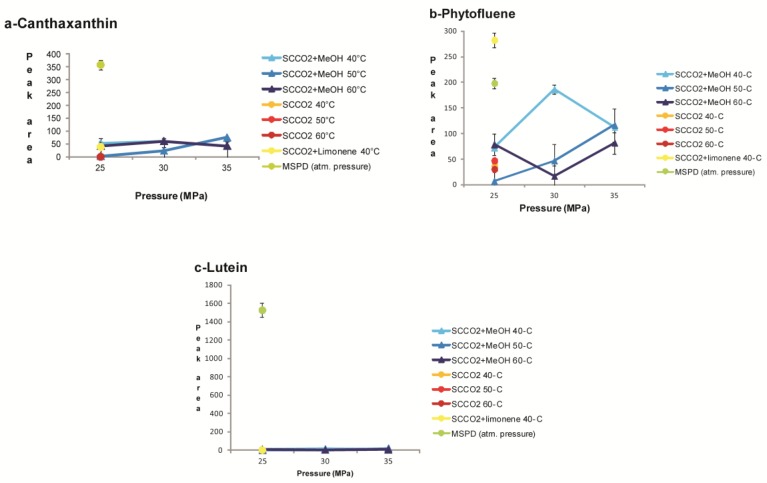
Extraction yields (average peak area ± standard error) of (**a**) canthaxanthin, (**b**) phytofluene, and (**c**) lutein obtained with SFE and MSPD.

Moreover, we investigated the effect of modifiers such as methanol (5% *v*/*v*) and limonene (5% *v*/*v*), on the composition of the extracts. As can be seen in Figure 1 and Figure 2, the extractions carried out with the addition of limonene did not show a qualitative or quantitative improvement in the composition of the extract, except for the best extraction of phytofluene (a non-polar compound structurally very similar to limonene), while as expected, the addition of methanol allowed a better recovery of all the more polar carotenoids.

#### 2.3.2. SCCO_2_ Extraction of Fat-Soluble Vitamins and Carotenoids from T. Obliquus in Comparison with MSPD 

**Figure 3:**
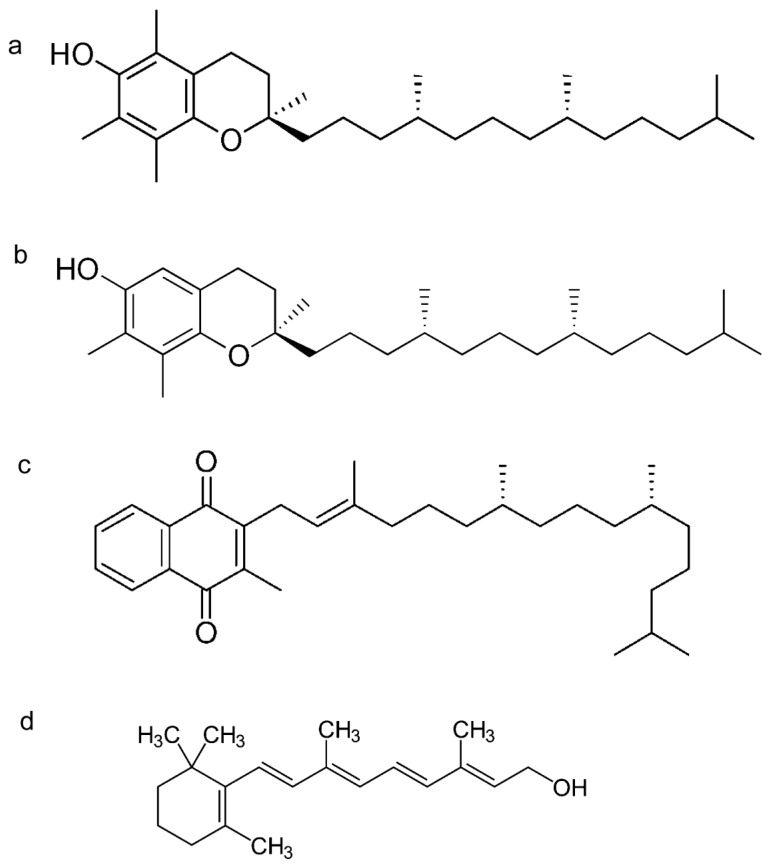
Chemical structures of: (**a**) alpha tocopherol, (**b**) gamma-tocopherol, (**c**) phylloquinone, and (**d**) retinol.

**Figure 4:**
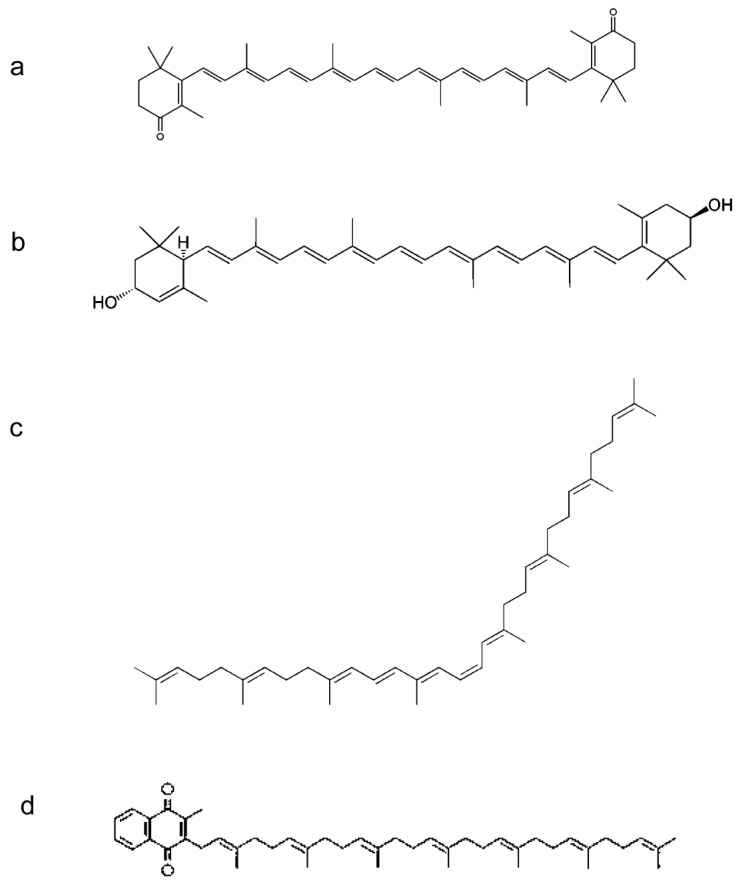
Chemical structures of: (**a**) canthaxanthin, (**b**) lutein, (**c**) phytofluene, and (**d**) menaquinone-7.

**Figure 5:**
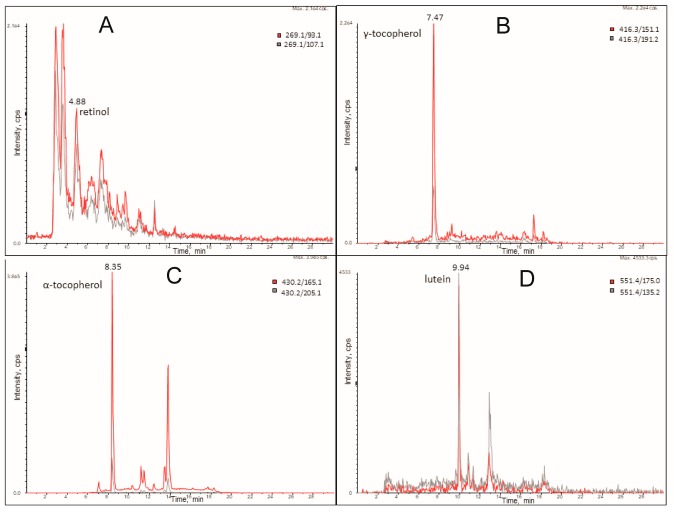
LC-MRM profile of retinol (**A**), gamma-tocopherol (**B**), alpha tocopherol (**C**) and lutein (**D**) extracted from a microalga sample by SFE. Extraction conditions: P_CO2_ = 35 MPa, T_CO2_ = 40 °C, 5% MeOH.

**Figure 6:**
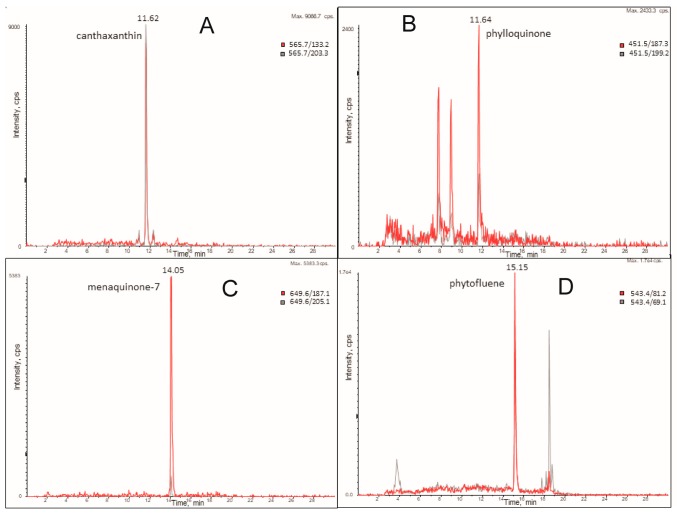
LC-MRM profile of canthaxanthin (**A**), phylloquinone (**B**), menaquinone-7 (**C**), and phytofluene (**D**) extracted from a microalga sample by SFE. Extraction conditions: A, B and D: P_CO2_ = 35 MPa, T_CO2_ = 40 °C, 5% MeOH. C: P_CO2_ = 10 MPa, T_CO2_ = 40 °C.

The supercritical fluid extraction of micronutrients from algal biomass in comparison with the solid/liquid extraction methodology is reported in Figure 1 and Figure 2. We optimized the extraction conditions in SCCO_2_ for the following compounds: α-tocopherol, canthaxantin, γ-tocopherol, lutein, phylloquinone, phytofluene, retinol, and menaquinone-7, whose structures are shown in Figure 3 and Figure 4. In Figure 5 and Figure 6, the mass spectra of each extracted compound are reported.

As reported in Figure 1a at P_CO2_ 30 MPa and T_CO2_ 40 °C with the addition of MeOH, the concentration of α-tocopherol detected in the extract was 61.8 μg/mL. The extraction with organic solvents allowed a lower recovery of α-tocopherol, with a concentration of 27.7 μg/mL. The molecular structure of α-tocopherol contains an oxygen atom that makes the molecule more polar than the carotenoids: this explains its higher SF extraction when adding MeOH as a cosolvent for CO_2_. 

Regarding alpha tocopherol extraction from different types of algae, a strong variability of its content has been reported in the literature and often very close taxa have very different alpha tocopherol contents. Such results point out the importance of growth conditions for obtaining higher quantities of this compound [32]. On this basis, it is very difficult to find useful extraction data to compare the efficiency of the extraction technique used.

Canthaxanthin is co-extracted with SCCO_2_ in the same experimental conditions (Figure 2a). The extraction yields, however, were not quantitatively comparable with those obtained by MSPD. It is most likely that the chemical structure of canthaxanthin, which contains two carbonyl groups, makes it very poorly soluble in supercritical CO_2_ (even at high density and in the presence of methanol). Indeed, the recovery of canthaxanthin in CO_2_-only extraction was below the limit of detection, while better results were obtained by increasing the polarity of the solvent phase with the addition of MeOH.

Phylloquinone contains two carbonyl groups together with the presence of a long hydrocarbon chain in its molecular structure. This feature can be responsible for a greater solubility in supercritical CO_2_ than in organic solvent mixtures. In the case of phylloquinone, the best extraction conditions were obtained at CO_2_ pressure values of 25 MPa and T = 60 °C in the presence of MeOH (Figure 1b).

γ-tocopherol, one of the eight vitamers of vitamin E, showed a better extraction profile with SCCO_2_, and the addition of MeOH at a temperature of 40 °C and pressure of both 35 and 30 MPa, produced quantitatively higher extraction yields than those obtained with the MSPD technique (Figure 1c). α-tocopherol and γ-tocopherol are structurally similar molecules, and despite being extracted under the same experimental conditions, their recovery was different due to the different relative quantities contained in the microalgae.

Retinol, or vitamin A, as a metabolite of provitamin A carotenoids, could be formed during the extraction procedure by increasing the extraction temperature. In fact, in almost all the SCCO_2_ extraction conditions tested, a larger recovery of this vitamin was observed when compared to the MSPD technique (Figure 1d).

Phytofluene, a colorless carotenoid precursor, has a structure with 40 carbon atoms and five conjugated double bonds. It showed a better recovery profile with the MSPD technique compared to SFE, although the addition of limonene allowed a better extraction of this carotenoid with the SFE technique (Figure 2b). This may be due to its remarkably apolar structure, while further degradation occurs at temperatures above 40 °C and at higher CO_2_ pressures.

Lutein, known as E161b in the European codification of food additives, was extracted in more significant amounts with the MSPD technique than with SCCO_2_ (Figure 2c). Lutein contains two hydroxyl groups within the molecule and is a very polar compound. It is partially recovered in SFE extractions with the addition of MeOH, but in lower relative yields.

Finally, extractions on the microalgae carried out in SCCO_2_ at a pressure of 10 MPa and T = 40 °C allowed the selective extraction of menaquinone-7 (Figure 6), which was not detected in the MSPD extractions. SCCO_2_ extractions at higher pressures and temperatures did not show the presence of menaquinone-7, confirming the hypothesis that it could be chemically degraded at high CO_2_ pressure or temperature values.

Menaquinone-7, like the other menaquinones, has a bacterial origin. It is not usually synthesized from algae, although in the literature, its presence has been hypothesized in the microalgae of the genus *Scenedesmus* [33]. Moreover, the microalgae used in this study were grown in a non-sterile environment, therefore they may have contained the products of a unique system formed by the microbiota in symbiosis with microalgae [34]. It has been proven that several microalgae species cannot survive without such associated bacteria because these latter furnish essential vitamins (such as vitamin B_12_) to the microalgae [35]. 

Our results demonstrate that SFE with SCCO_2_ is a green method for the extraction of high purity thermolabile compounds such as carotenoids. However, the yield of polar carotenoids, as reported in the literature, is often low [36]. By optimizing some key parameters (use of entrainers), it is possible to improve the solubility of more polar analytes in SCCO_2_. Table 1 reports a comparison of the obtained SFE extraction yields based on the peak areas obtained from the mass spectra with those obtained with MSPD. By varying the SFE conditions, we were able to obtain comparable and sometimes higher extraction yields than MSPD for alpha and gamma tocopherol, canthaxanthin, phylloquinone, phytofluene, retinol, and menaquinone-7.

## 3. Materials and Methods 

### 3.1. Biomass Production

A strain of the microalgae *Tetradesmus obliquus* was maintained in the laboratory under phototrophic conditions as previously described [36]. *T. obliquus* is generally known as *Scenedesmus obliquus*, but it has been recently reclassified by Wynne and Hallan [37].

The biomass used for the extraction tests was produced by diluting 1 to 10 (*v*/*v*) microalgae from the maintenance flasks in two column photobioreactors (ø = 9 cm, h = 65 cm) with the cultivation medium. The initial biomass concentration was 0.05 g/L. The cultivation medium used was a “tap water based medium”, which is a cultivation medium obtained by adding NaNO_3_ and K_2_HPO_4_ to the local tap water; its exact chemical composition has been described in a previous work [38,39]. The photobioreactors were maintained under 24 h/24 constant illumination at 100 µmol m^−2^s^−1^, by means of cool-white florescent lamps and constant air feeding (0.5 L/min) at a room temperature of 27 ± 3 °C. After 15 days of cultivation, the produced microalgae biomass was harvested by centrifugation at 1370× *g* for 5 min and then the obtained pellet was freeze dried.

### 3.2. Chemicals and Solvents 

Methanol, ethanol, 2-propanol, hexane, and acetone (HPLC grade) were purchased from Sigma (St. Louis, MO, USA). Dichloromethane and acetonitrile (analytical grade) were obtained from Chromasolv (Barcelona, Spain). Diatomaceous earth SPE-ED MATRIX 38 was purchased from Applied Separations (Allentown, PA, USA). Syringe-like polypropylene tubes (i.d. 26 mm, 75 mL capacity) and polyethylene frits were obtained from Alltech (Deerfield, IL, USA).

The following standards were purchased from Aldrich-Fluka-Sigma Chemical (St. Louis, MO, USA): retinol, ergocalciferol, δ-tocopherol, β-tocopherol, γ-tocopherol, cholecalciferol, α-tocopherol, menaquinone-4, menaquinone-7, phylloquinone, all-trans-lutein, all trans-zeaxanthin, all-trans-β-cryptoxanthin, and all-trans-β-carotene. Standards of α-tocotrienol, β-tocotrienol, δ-tocotrienol, and γ-tocotrienol were bought from LGC Standards (Middlesex, U.K.). Standards of 15-cis-phytoene, all-trans-phytoene, all-trans-phytofluene, 13-cis-β-carotene, 9-cis-β-carotene, all-trans-ζ-carotene, all-trans-γ-carotene, all-trans-lycopene, and 5-cis-lycopene were purchased from CaroteNature GmbH (Ostermundigen, Switzerland). All chemicals had a purity grade of >97%.

### 3.3. Biomass Pretreatment

The biomass was freeze dried and stored at −20 °C. Before extraction, the biomass was manually ground into a fine powder. When diatomaceous earth was used, it was mixed with the ground biomass at a 1:10 *w*/*w* ratio (algae:diatomaceous earth).

### 3.4. MSPD Extraction

The results obtained by SFE were compared with those from the MSPD extraction. The last one was performed as follows: 200 mg of sample (biomass and diatomaceous earth 1:10 *w*/*w*) was ground with a pestle into a ceramic mortar until an evenly colored powder was obtained. Subsequently, this powder was used for filling a syringe-like polypropylene tube previously prepared with a first layer of C18 sorbent (0.4 g). The resultant chromatographic bed was held among two polyethylene frits. Vacuum-assisted elution was conducted with 15 mL of methanol, 5 mL of 2-propanol, and 20 mL of hexane by collecting the analytes into a 50 mL falcon. Samples were then centrifuged at 6000 rpm for 10 min. The supernatant was poured out into a glass tube with a conical bottom (i.d. 2 cm) and evaporated up to dryness under a gentle flow of nitrogen in a water bath kept at 25 °C. Finally, the dry extract was dissolved in 2 mL of ethanol, sonicated for 2 min, and passed through a PTFE 0.45 μm filter. Forty microliters were injected into the chromatographic column for the LC-MS analysis. 

### 3.5. Supercritical Fluid Extraction

Supercritical fluid extractions (SFE) were performed on a SFE 300 analytical extractor manufactured by Carlo Erba Instruments. A scheme of the extraction apparatus is shown in Figure 1. The extractions took place in a metal tubular reactor of 1 cm^3^ where the samples were introduced. Cooled CO_2_ was fed into the high-pressure reactor, pressurized at the desired target pressure by a syringe pump, and heated to the desired temperature with a system of recirculating air in the thermostated chamber where the reactor was located. In all of the experiments, a static extraction was followed by a 10-min dynamic extraction performed through the depressurization of SCCO_2_ in 2 mL of ethanol.

For each extraction experiment, 200 mg of ground powder (containing biomass and diatomaceous earth 1:10 *w*/*w*) were placed in the extraction cell. The operating parameters were varied in the pressure range of 10–35 MPa and in the temperature range of 40–60 °C. A series of extractions was also performed in the presence of a modifier by adding 5% (*v*/*v*) methanol (T = 40, 50, and 60 °C; P = 25, 30, and 35 MPa) or limonene (T = 40 °C; P = 30 MPa) to the sample inside the extraction cell. 

### 3.6. Mass Spectrometry Experiments 

Analytes were detected by a 4000 Qtrap (AB SCIEX, Foster City, CA, USA.) mass spectrometer equipped with an atmospheric pressure chemical ionization (APCI) probe on a Turbo V source. A positive ionization mode was used, setting a needle current (NC) of 3 μA and a probe temperature of 450 °C. High-purity nitrogen was used as the curtain (40 psi) and collision (4 mTorr) gas, whereas air was the nebulizer (55 psi) and makeup (30 psi) gas. The preliminary calibration of Q1 and Q3 mass analyzers was conducted by infusing a polypropylene glycol solution at 10 μL/min. The unit mass resolution was established by maintaining a full width at half-maximum (fwhm) of approximately 0.7 ± 0.1 unit in each mass-resolving quadrupole. APCI−Q1−full scan spectra and product ion scan spectra of the analytes were acquired by working in flow injection analysis (1−10 ng injected, 1 mL/min flow rate).

### 3.7. Liquid Chromatography

Liquid chromatography (LC) was performed by a micro HPLC series 200 (PerkinElmer, Norwalk, CT, USA.) equipped with an autosampler, vacuum degasser, and column chiller. Analytes were separated on a ProntoSIL C30 column (4.6 mm × 250 mm, 3 μm) from Bischoff Chromatography (Leonberg, Germany), protected by a guard C30 column (4.0 mm × 10 mm, 5 μm), under non aqueous-reversed phase (NARP) conditions at 19 °C. The elution profile by using methanol (phase A) and 2-propanol/hexane (50:50, *v*/*v*; phase B) was as follows: 0−1 min, 0% B; 1−15 min, 0−75% B; 15−15.1 min, 75−99.5% B; and 15.1−30.1 min, 99.5% B. The mobile phase was entirely introduced into the MS detector at a flow rate of 1 mL/min. Phase B was also used to wash the autosampler injection device.

The separation and detection of MK-7 were confirmed by using a specific chromatographic method with increased efficiency in separating vitamin K homologues from interfering compounds. This method differs from the previous one for the use of two reversed-phase columns connected in series (SUPELCOSILTM C18, 4.6 mm × 50 mm, 5 mm, Supelco–Sigma–Aldrich, Bellefonte, PA, USA; and Alltima C18, 4.6 mm × 250 mm; 5 mm, Alltech, Deerfield, IL, USA). 

## 4. Conclusions

In this study, the influence of parameters such as pressure, temperature, and the addition of a polar co-solvent on the SFE yields of carotenoids and fat-soluble vitamins from *T. obliquus* biomass was studied. The optimized extraction conditions revealed the possibility to substantially increase the yields of some compounds with respect to conventional solid–liquid extraction. In particular, by varying the SFE polarity, we were able to obtain comparable and sometimes higher extraction yields than MSPD for many low or medium-polar carotenoids and vitamins. We also obtained a remarkable selectivity (at 10 MPa and 40 °C) for the extraction of the compound menaquinone-7, whose extraction has been rarely achieved by using traditional procedures.

## Figures and Tables

**Table 1:** The SFE extraction yields of alpha tocopherol, phylloquinone, gamma-tocopherol, retinol, canthaxanthin, phytofluene, and lutein using different experimental conditions. The values reported are expressed as percentages of MSPD extraction yields ± standard error.

Compound	SCCO_2_ 25 MPa			SCCO_2_ + limonene	SCCO_2_ + MeOH 25 MPa			SCCO_2_ + MeOH 30 MPa			SCCO_2_ + MeOH 35 MPa		
	40 °C	50 °C	60 °C		40 °C	50 °C	60 °C	40 °C	50 °C	60 °C	40 °C	50 °C	60 °C
Alpha-tocopherol	(77.71 ± 4.95)%	(97.3 ± 3.59)%	(71.12 ± 3.91)%	(78.73 ± 3.28)%	(86.25 ± 5.16)%	(2.88 ± 2.76)%	(156.95 ± 5.31)%	(200.15 ± 4.64)%	(122.39 ± 4.61)%	(133.44 ± 5.45)%	(180.73 ± 5.33)%	(185.79 ± 3.78)%	(139.39 ± 3.74)%
Phylloquinone	(38.83 ± 4.61)%	(86.50 ± 4.31)%	(34.77 ± 4.31)%	(21.45 ± 4.15)%	(262.14 ± 5.16)%	0	(302.74 ± 5.89)%	(154.46 ± 5.72)%	(71.67 ± 6.11)%	(50.31 ± 6.73)%	(151.81 ± 5.63)%	(174.76 ± 6.28)%	(108.56 ± 6.45)%
Gamma-tocopherol	(41.42 ± 3.76)%	(43.26 ± 3.73)%	(21.77 ± 5.71)%	(86.73 ± 3.82)%	(69.74 ± 3.16)%	(4.09 ± 3.16)%	(115.46 ± 3.81)%	(128.90 ± 3.45)%	(65.30 ± 3.18)%	(74.72 ± 3.62)%	(137.43 ± 3.92)%	(115.52 ± 3.75)%	(88.98 ± 3.49)%
Retinol	(81.44 ± 3.18)%	(212.56 ± 3.47)%	(62.03 ± 3.94)%	0	(332.72 ± 2.58)%	0	(213.75 ± 5.76)%	(543.00 ± 5.73)%	(430.97 ± 8.56)%	(317.32 ± 4.61)%	(213.00 ± 3.94)%	(192.93 ± 6.31)%	(94.78 ± 4.72)%
Canthaxanthin	0	0	0	(8.09 ± 2.98)%	(11.02 ± 3.11)%	(0.56 ± 0.44)%	(9.16 ± 3.26)%	(12.99 ± 3.45)%	(4.99 ± 2.31)%	(12.99 ± 4.23)%	(9.25 ± 3.62)%	(16.14 ± 3.15)%	(9.25 ± 3.52)%
Phytofluene	(18.24 ± 5.64)%	(23.50 ± 4.25)%	(15.21 ± 2.58)%	(142.65 ± 7.51)%	(36.43 ± 4.39)%	(94.04 ± 3.98)%	(56.80 ± 4.58)%	(3.34 ± 2.13)%	(23.50 ± 8.57)%	(58.92 ± 8.63)%	(39.46 ± 8.91)%	(8.44 ± 7.32)%	(41.13 ± 8.69)%
Lutein	0	0	0	0	(0.74 ± 0.53)%	0	(0.70 ± 0.45)%	(1.03 ± 0.74)%	(0.29 ± 0.25)%	(0.22 ± 0.20)%	(0.67 ± 0.64)%	(1.25 ± 0.50)%	(0.40 ± 0.40)%
